# O304 alleviates abdominal aortic aneurysm formation via AMPK/mTOR/MMP pathway activation

**DOI:** 10.3389/fphar.2024.1457817

**Published:** 2024-11-29

**Authors:** Daohan Sun, Yaming Du

**Affiliations:** Department of Vascular Surgery, The First Affiliated Hospital of Jinzhou Medical University, Jinzhou, Liaoning, China

**Keywords:** abdominal aortic aneurysm, contractile vascular smooth muscle cell, AMPK, O304, mTOR

## Abstract

**Background:**

Abdominal aortic aneurysm (AAA) rupture is a significant cause of mortality in the elderly population. Despite experimental models identifying promising pharmacological therapies, there is still a lack of pharmacological interventions for AAA prior to surgery. This study aims to evaluate the regulatory role of the novel adenosine monophosphate-activated protein kinase (AMPK) agonist O304 in AAA formation and explore its underlying molecular mechanisms.

**Methods:**

We evaluated the expression of AMPK signaling pathway components and contractile vascular smooth muscle cell (VSMC)-related genes in AAA samples from mice using reverse transcription-quantitative polymerase chain reaction (RT-qPCR). We evaluate the TGF-β expression by western blotting and RT-qPCR and TGF-β concentration in blood by ELISA. We developed an *in vitro* model of transforming growth factor-β (TGF-β)-induced VSMC phenotypic switching. After treatment with O304, we analyzed the expression of contractile genes and proteins in VSMCs by immunofluorescence and Western blotting. We also evaluated the expression of AMPK signaling pathway components and matrix metalloproteinases by western blotting and immunofluorescence analysis. We established a mouse model of AAA to evaluate the impact of O304 on aneurysm diameter and blood pressure, analyzed VSMC phenotypic switching through immunofluorescence analysis, and assessed the regulatory effects of O304 on AMPK signaling in the mouse model of AAA by Western blotting.

**Results:**

AMPK signaling pathway components and contractile genes in VSMCs were downregulated in mouse AAA samples, underscoring the crucial role of AMPK signaling in VSMC phenotypic switching. In the TGF-β-induced model of VSMC phenotypic switching, O304 activated AMPK signaling and prevented VSMC phenotypic switching from the contractile to the synthetic phenotype. Moreover, O304 significantly activated AMPK signaling, increased the proportion of contractile VSMCs, and reduced AAA formation and blood pressure in the mouse model of AAA.

**Conclusion:**

During AAA development, VSMCs transitioned from the contractile to the proliferative phenotype, a process that has previously been associated with AMPK pathway inhibition. O304, an AMPK agonist, activated the AMPK pathway, preventing VSMC phenotypic switching and inhibiting AAA formation. These findings highlight the therapeutic potential of targeting the AMPK pathway in AAA.

## Introduction

Abdominal aortic aneurysm (AAA) rupture is a significant cause of mortality among the elderly population, with a mortality rate of up to 65.9% (Golledge, 2019). AAA rupture results from gradual weakening of the aortic wall, leading to dilation and eventual rupture of the aorta, which is often accompanied by intense inflammation. Key cardiovascular risk factors, such as hypertension, tobacco use, and hyperlipidemia, contribute to AAA development (Chaikof et al., 2018; Kleinloog et al., 2018) While Several pharmacological strategies have shown therapeutic potential in experimental models; however, none have been successfully translated into clinical practice, leaving surgical repair as the primary treatment for advanced AAA. The lack of pharmacological interventions that can be implemented prior to surgery for AAA rupture underscores the pressing need for novel drug-based treatments (Sakalihasan et al., 2005).

The pathogenesis of AAA involves various pathological processes, including leukocyte infiltration into the vessel wall (Tulamo et al., 2018), neovascularization, loss of vascular smooth muscle cells (VSMCs), and extracellular matrix (ECM) degradation (Schiffrin, 2012). VSMCs are crucial components of the vascular wall. They exhibit high plasticity and the ability to transition between the contractile and synthetic phenotypes in response to various stimuli (Bennett et al., 2016; Brown et al., 2018; Krings et al., 2011). In healthy arteries, VSMCs predominantly adopt the contractile phenotype and are located in the tunica media, where they are responsible for vascular contraction and ECM deposition, which are important for arterial compliance and elastic recoil (Lacolley et al., 2017). VSMC phenotypic switching from the contractile to the synthetic phenotype is a key factor in AAA development (Starke et al., 2014). Contractile VSMCs maintain vascular homeostasis, while their transition to the synthetic phenotype under pathological conditions promotes inflammation and matrix metalloproteinase (MMP) production, both of which are closely linked to AAA progression (Frösen, 2014; Owens et al., 2004; Alexander and Owens, 2012). Although research efforts are ongoing to elucidate the mechanisms underpinning AAA, effective pharmacological agents that can prevent aneurysm formation or delay AAA rupture remain to be identified. Nevertheless, targeting VSMC phenotypic switching may represent a novel therapeutic strategy for AAA.

Adenosine monophosphate-activated protein kinase (AMPK) is a key regulator of cellular metabolic homeostasis and diverse biological processes. AMPK, which is a sensor of cellular energy status, plays a pivotal role in modulating energy metabolism, cellular growth, and autophagy (Jeon, 2016). AMPK also regulates inflammation, angiogenesis, and ECM dynamics (Zhou et al., 2001). Clinical investigations have revealed a notable decrease in phosphorylated AMPK (p-AMPK) within AAA tissues, suggesting downregulation of the AMPK signaling pathway in human AAA (Li et al., 2019; Lin et al., 2021). This dysregulation suggests a possible contribution of AMPK signaling alterations to AAA development and progression.

In this study, we utilize a novel AMPK agonist, O304, to activate the AMPK pathway. Through a combination of *in vitro* and *in vivo* experiments, we aim to elucidate the potential of O304 to inhibit AAA formation and unravel the complex mechanistic pathways regulated by AMPK in AAA. Understanding the intricate interactions of AMPK signaling in AAA pathogenesis may help to identify novel pharmacological candidates.

## Methods

### Animal experiments

All animal experiments were conducted in strict accordance with the guidelines outlined in the Animal Research: Reporting of *In Vivo* Experiments (ARRIVE) 2.0 guidelines. The protocols and procedures involving animals were performed in compliance with the Guide for the Care and Use of Laboratory Animals issued by the US National Institutes of Health (eighth Edition, 2011). ApoE-deficient (ApoE^−/−^) male mice aged 8–11 weeks were obtained from the Shanghai Model Organisms Center (Shanghai, China). The mice were housed individually under specific pathogen-free conditions in ventilated cages maintained at a temperature of 20°C–24°C with a humidity of 50%–60%. The cages were equipped with a ventilation system that provided 60 air exchanges per hour to ensure optimal air quality and comfort for the animals. The mice were subjected to a standard diurnal 12-hour light/dark cycle with *ad libitum* access to food and water. Environmental enrichment measures were implemented to promote the wellbeing of the animals, including the provision of nesting material, PVC pipes, and shelter. All equipment and materials, including the cages, lids, feeders, bottles, bedding, and water, underwent autoclaving before use to maintain sterile conditions and ensure the health and safety of the animals throughout the experiment.

### Mouse model of AAA

We randomly assigned the mice to different groups using computer-generated random numbers to ensure unbiased group allocation. The investigators were blinded to the group assignment and evaluated the outcomes after treatment administration to minimize potential biases. The mouse model of AAA was established as previously described (Yu et al., 2023; Zhang et al., 2023). Briefly, 6-week-old mice were allowed *ad libitum* access to a high-fat diet containing milk fat 15% with 1.2% cholesterol made into pellet form for 4 weeks. Subsequently, osmotic pumps containing angiotensin II (Sigma-Aldrich, St. Louis, MO, US) were implanted through a small incision in the back, and angiotensin II (1,000 ng/kg/min) was released via the pumps for 28 days. For drug administration, we selected a dosing regimen of 200 mg/kg via oral administration, informed by the dosing protocols of O304 utilized in other models (Das et al., 2019). Specifically, 160 mg of O304 (Selleck, China) was initially dissolved in 8 mL of DMSO to create a stock solution with a concentration of 20 mg/mL. This stock solution was then diluted with PBS to prepare a working solution at a concentration of 4 mg/mL. Consequently, each mouse, weighing 20 g, received 1 mL of the working solution daily, achieving a dosage of 200 mg/kg. Furthermore, each mouse was treated once every 3 days post-surgery, continuing for a total duration of 28 days, which amounted to 7 doses overall. Mice in the control group underwent the same surgical procedures for AAA induction as the treatment group, including anesthesia and implantation of the mini-pump for angiotensin II infusion. However, instead of receiving O304, mice in the control group were administered an equal volume of phosphate-buffered saline (PBS) as a vehicle control. This design ensures that any differences between the control and O304-treated groups can be attributed to the effects of O304, rather than procedural factors or vehicle administration. The sham group underwent the same surgical interventions as the other groups, such as anesthesia and surgical exposure of the abdominal region. However, unlike the control and treatment groups, the sham group did not receive angiotensin II infusion or any other agents. This group served as a baseline reference for evaluating the physiological effects of the surgery itself, allowing us to distinguish between changes caused by the AAA-inducing agent and the surgical procedure. On day 28 after implantation, the diameter of the abdominal aorta was measured. The aorta was then dissected for immunofluorescence staining and Western blotting to assess the effects of O304.

### Blood pressure measurement

Systolic blood pressure (SBP), and diastolic blood pressure (DBP) were measured prior to mini-pump implantation (day 0) and then once weekly for 4 weeks post-implantation using a non-invasive tail-cuff system (Softron, Japan) (Guo et al., 2023; Lin et al., 2023). The tail-cuff plethysmography technique allowed for accurate and repeatable measurements in conscious, restrained mice. Measurements were taken in a controlled, quiet environment, and the tail cuffs were warmed to 34°C to promote proper blood flow. Multiple readings (at least five per session) were recorded for each mouse, and the average SBP, DBP, and HR values were calculated for each time point. Data collection was carried out consistently at the same time of day to avoid fluctuations due to circadian rhythm. Blood pressure and heart rate trends were compared between the AAA model and O304-treated mice throughout the 28-day period to assess the impact of angiotensin II and O304 treatment on blood pressure parameters.

### VSMC culture

Human aortic smooth muscle cells (ScienCell Research Laboratories, Carlsbad, CA, United States) were cultured in smooth muscle cell medium (ScienCell Research Laboratories) supplemented with 2% fetal bovine serum (FBS). The cells were maintained in a humidified incubator at 37°C with 5% CO_2_. Before cell seeding, the culture dishes were pretreated with poly-L-lysine to enhance cell adhesion. For experimental purposes, cells from passages 3–5 were utilized to ensure consistency and reliability in the subsequent analyses.

### Induction of VSMC phenotypic switching

Based on previous literature and the methods established in our laboratory, we cultured VSMCs and induced phenotypic switching (Li S. et al., 2020). The VSMCs were rapidly thawed from liquid nitrogen and added to prewarmed culture medium for subsequent preculturing at 37°C in a humidified incubator with 5% CO_2_ in medium containing 2% FBS. Upon reaching 80%–90% confluence, at which a dense and uniform cell layer was established, the cells were passaged by gentle washing with DPBS and digestion with 0.25% Trypsin-EDTA at 37°C. Digestion was halted upon observing cell detachment under a microscope, followed by neutralization with prewarmed medium. The cells were counted using a hemocytometer and redistributed into new culture bottles as required for experimentation.

To induce phenotypic switching, we prepared the culture medium by adding 10% FBS to Dulbecco’s Modified Eagle Medium supplemented with 10 mM transforming growth factor-β (TGF-β). The solution was thoroughly mixed to ensure even distribution of the TGF-β, which was subsequently used for treatment. The treatment group received 50 nM O304, while the control group underwent an identical procedure except that the mice in the control group were treated with an equal volume of PBS. The culture medium was replaced by gently rinsing the VSMCs with DPBS to remove the original medium and adding freshly prepared medium containing TGF-β. The culture bottles were placed in a humidified incubator at 37°C with 5% CO_2_. Regular medium changes were performed to ensure nutrient sufficiency and maintenance of an FBS concentration of 2%. The cell morphology was monitored under a microscope to ensure a typical smooth muscle cell appearance. Immunocytochemistry and other identification methods were utilized to observe normal cell marker expression and to confirm the identity of the cells as aortic smooth muscle cells.

### Immunofluorescence staining

The mice were sacrificed 28 days after surgery, and infrarenal segments of the aorta were harvested. According to published literature and the methods established in our laboratory (Zhu et al., 2022), we performed immunofluorescence staining. First, VSMCs were cultured and seeded into 24-well plates or four-chamber slides to ensure an even cell distribution for subsequent cell slide preparation. Upon reaching 85%–90% confluence, the cells were gently washed with PBS to remove residual culture medium. The cells were fixed with 4% paraformaldehyde at room temperature for 15 min to preserve the integrity of the cell structure. After fixation, residual paraformaldehyde was washed off using PBS three times for 5 min each time to prevent interference in the subsequent steps. The cell membranes were permeabilized with 0.4% Triton X-100 at room temperature for 10 min to enhance antibody penetration. Residual Triton X-100 was washed off with PBS three times for 5 min each time. The cells were then blocked with 5% bovine serum albumin (BSA) in PBS at room temperature for 60 min to reduce non-specific binding. After blocking, the cells or tissue sections were incubated overnight at 4°C with anti-α-smooth muscle actin (SMA) (Abcam, Shanghai, China), anti-Ki67 (Abcam), anti-SM22 (Abcam), anti-elastin (Abcam), anti-MMP2 (proteintech), anti-MMP3 (proteintech), or anti-MMP9 (proteintech) at 4°C. The next day, the cells were washed three times with PBS for 5 min each time to remove any unbound primary antibody. Subsequently, secondary antibodies diluted in PBS were incubated at room temperature for 2 h. After incubation, the cells were washed three times with PBS for 5 min each time to remove any unbound secondary antibody. Finally, the cells were washed with deionized water to remove any residual ion contamination and mounted with a mounting medium containing DAPI to label the cell nuclei. Images were captured using a fully automated fluorescence microscope (Leica). Image analysis, including quantitative measurement of fluorescence intensity, was performed using Lax and Image J software simultaneously.

### ELISA

The concentration of TGF-β in mouse serum was measured using a commercially available enzyme-linked immunosorbent assay (ELISA) kit (Beyotime, China), following the manufacturer’s protocol. Briefly, whole blood was collected from mice via retro-orbital bleeding into tubes without anticoagulants to allow coagulation. The samples were left at room temperature for 30 min to clot and then centrifuged at 1,500 × g for 15 min at 4°C to isolate the serum. Serum samples were aliquoted and stored at −80°C until analysis. Prior to the ELISA assay, the serum samples were thawed on ice and diluted according to the kit’s instructions to fall within the assay’s detection range. A standard curve was prepared using serial dilutions of recombinant TGF-β protein provided in the kit. Both the standards and serum samples were added to 96-well microplates pre-coated with an anti-TGF-β antibody and incubated for 2 h at room temperature to allow binding. After incubation, the wells were washed thoroughly with the provided wash buffer to remove unbound substances. A biotin-conjugated detection antibody specific to TGF-β was then added, followed by streptavidin-HRP (horseradish peroxidase) to amplify the signal. The plates were incubated for an additional hour, washed, and developed with the substrate solution. After 15–20 min of color development, the reaction was stopped using a stop solution, and the absorbance was read at 450 nm using a microplate reader. The TGF-β concentration in the serum samples was calculated by comparing the absorbance values to the standard curve. All samples were assayed in duplicate to ensure accuracy and the results were normalized to normal group.

### Protein extraction

Referring to previous literature and the methods established in our laboratory (Yu et al., 2022), cell and tissue protein extraction was performed. For tissue protein collection and lysis, the abdominal aorta was retrieved, rapidly washed, and added to RIPA lysis buffer containing protease and phosphatase inhibitors at a concentration of 100 mg/mL. The tissues were homogenized to ensure thorough disruption while maintaining ice-cold conditions to minimize protein degradation. Then, the tissue was lysed on ice for 40 min and centrifuged at 12,000 rpm for 20 min at 4°C. The supernatant containing the extracted protein was collected after discarding the precipitation. For cell protein collection and lysis, the cells were washed with prechilled PBS and treated with RIPA lysis buffer containing protease and phosphatase inhibitors. The cells were scraped off using a cell scraper, and the lysate was transferred to ice for 40 min for further lysis. After centrifugation at 12,000 rpm for 20 min at 4°C, the supernatant containing the cell proteins was collected after discarding the pellet. The protein concentration was quantified using the bicinchoninic acid (BCA) assay to ensure consistent protein concentrations among the samples. To achieve uniform concentrations, the protein supernatants from each group were mixed with 5 × loading buffer containing DTT, heated at 96°C in a metal bath for 10 min to denature the proteins, and cooled for long-term storage at −80°C for subsequent experiments.

### Western blotting

Based on previous literature and the methods established in our laboratory (Zhou et al., 2022), Western blotting was performed. First, proteins from the abdominal aorta tissues and VSMCs were carefully separated using a mixture of protease and phosphatase inhibitors under low-temperature conditions to maintain protein integrity. The protein concentrations were precisely determined using the BCA method (Thermo Fisher Scientific). The samples were diluted according to the protein concentration to ensure equal loading of the proteins for sodium dodecyl sulfate-polyacrylamide gel electrophoresis, followed by separation using a protein electrophoresis system (BioRad). The total protein was transferred onto polyvinylidene fluoride (PVDF) membranes using a semi-dry transfer system for subsequent immunoblotting analysis. The PVDF membranes were blocked with 5% BSA at room temperature for 2 h to reduce non-specific binding. Specific primary antibodies, including primary antibodies against α-SMA, colponin, SM22, t-AMPK, p-AMPK, mammalian target of rapamycin (mTOR), MMP2, MMP3, MMP9, TGF-β, and anti-glyceraldehyde-3-phosphate dehydrogenase (GAPDH), were incubated overnight with the membranes at 4°C, followed by incubation with horseradish peroxidase-conjugated secondary antibodies at room temperature for 2 h. The protein bands were visualized using an infrared imaging system, and quantitative analysis was performed using image analysis software to measure the relative expression of target proteins compared with the reference protein (GAPDH).

### RNA extraction and reverse transcription-quantitative polymerase chain reaction (RT-qPCR)

RNA extraction and RT-qPCR were performed as described previously (Pan et al., 2022). Total RNA was extracted from the cells and abdominal aorta tissues using TRIzol reagent. Then, complementary DNA (cDNA) synthesis was performed using the HiFiScript cDNA Synthesis Kit (CoWin Biosciences, Shanghai, China). The cDNA was amplified using the QuantStudio 7 Flex Real-time PCR system (Applied Biosystems, CA, United States) with SYBR Green Master Mix (Takara Bio Company, Shiga, Japan) according to the manufacturers’ instructions. *GAPDH* served as the internal control for normalization. The relative expression of each type of mRNA was determined using the 2^−ΔΔCT^ method.

### Statistical analysis

The data are presented as the mean ± standard error of the mean. The normality of the data distribution was assessed using the Shapiro–Wilk test. Differences between two mean values were identified using the Student’s t-test, while multiple comparisons were performed by analysis of variance with Tukey’s *post hoc* test. All statistical analyses were performed using SPSS software (version 18.0; IBM Corp., Armonk, NY, United States). *p* < 0.05 was considered statistically significant.

## Results

### AMPK and VSMC contractile genes increased in mice with AAA

**FIGURE 1:**
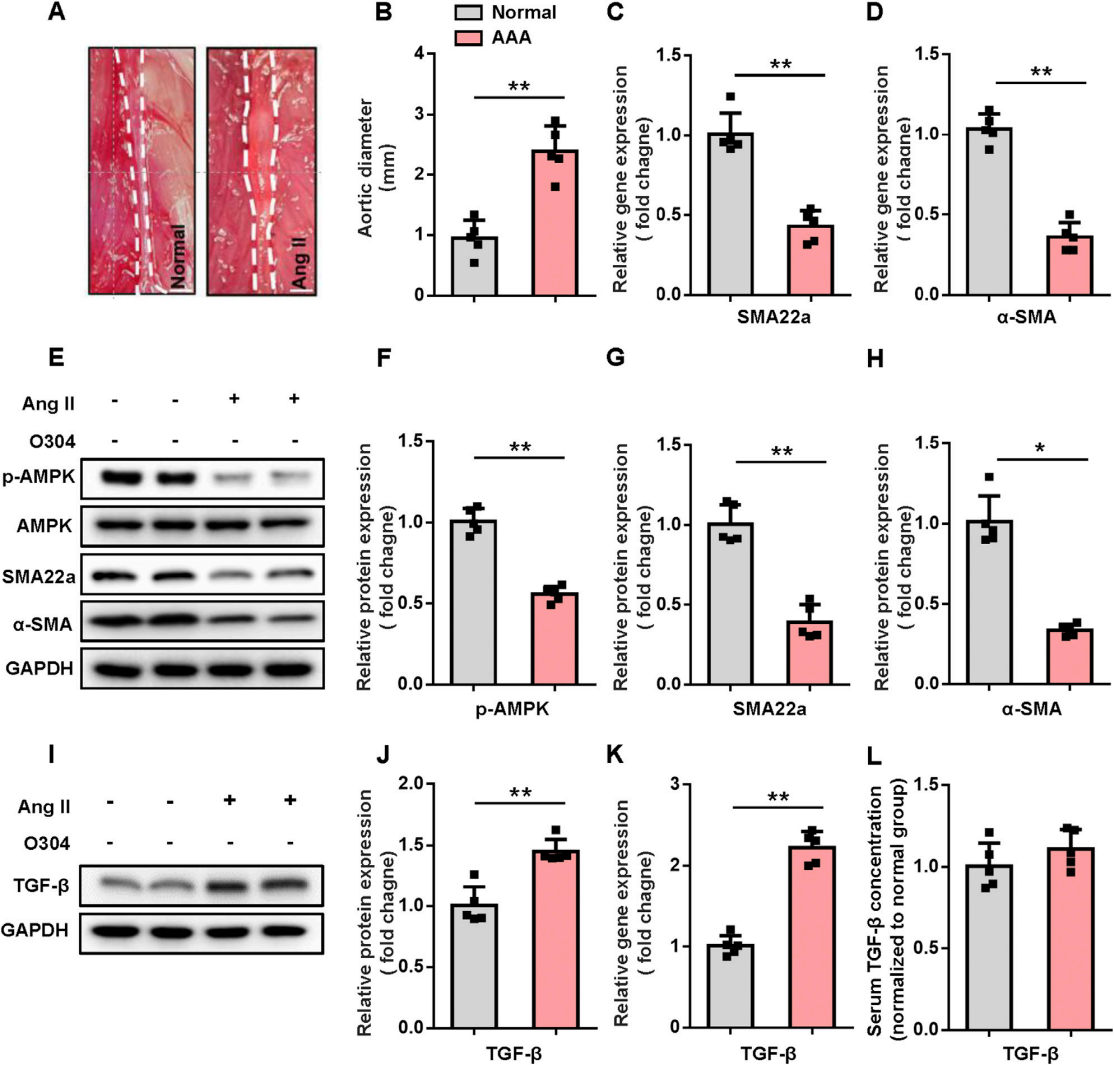
AMPK expression and contractile VSMC genes are upregulated in AAA. **(A–C)** Angiotensin II (1,000 ng/kg/min) was administered into the abdominal aorta via a micro-infusion pump for 4 weeks to establish a mouse model of AAA. **(A)** Representative image of the abdominal aorta after surgery. **(B)** Quantification of the aortic diameter. **(C, D)** RT-qPCR of contractile genes **(C)** SM22a and **(D)** α-SMA in VSMCs was conducted after surgery and normalized to GAPDH. **(E–H)** Western blotting was performed to assess the protein expression of AMPK signal pathway and contractile VSMCs marker. **(E)** Representative immunoblots. Full-length blots were presented in Supplementary Figure S2. Quantification of protein expression of **(F)** p-AMPK, **(G)** SM22a, and **(H)** α-SMA via densitometry analysis and normalization to GAPDH. **(I)** Western blotting was performed to assess the expression of **(J)** TGF-β. Quantification of protein expression via densitometry analysis and normalization to GAPDH. **(K)** RT-qPCR was performed to assess the gene expression of TGF-β. **(L)** Serum samples from sham and angiotensin II group mice were analysed using enzyme-linked immunosorbent assay (ELISA) to determine the levels of TGF-β and normalized to normal group. n = 4 – 5 independent experiments. Significance was evaluated using the Student’s t-test in **(B–D)**, **(F–H)**, and **(J–L)** *p < 0.05 and **p < 0.01. AAA, abdominal aortic aneurysm; AMPK, adenosine monophosphate-activated protein kinase; GAPDH, glyceraldehyde-3-phosphate dehydrogenase; RT-qPCR, reverse transcription-quantitative polymerase chain reaction; VSMC, vascular smooth muscle cell; ELISA, enzyme-linked immunosorbent assay.

To determine the relationship between contractile VSMCs and AAA, we constructed the mouse model of AAA and evaluated the diameter of the abdominal aorta 28 days after surgery. AAA was defined as an increase of >50% in the aortic diameter from baseline according to previous literature. A significant increase in the diameter of the abdominal aorta confirmed that the AAA model had been successfully established (Figures 1A, B). RT-qPCR was performed to measure gene expression in the mouse model of AAA. The results revealed that the genes associated with contractile VSMCs, including α-SMA, and SM22a were downregulated in the AAA group compared with the control group (Figures 1C, D). Moreover, we also measured protein expression associated with contractile VSMCs and AMPK via Western blotting. The results revealed a marked downregulation of α-SMA and SM22α, accompanied by a corresponding decrease in phosphorylated AMPK levels (Figures 1E–H; Supplementary Figure S1). These results suggested a critical link between the AMPK signaling pathway, VSMC phenotype, and AAA development, demonstrating that contractile VSMC phenotypic switching is an important pathological process in AAA that is accompanied by downregulation of AMPK signaling components. Despite ongoing debate regarding the role of TGF-β in AAA pathogenesis, studies have shown elevated levels of TGF-β in both human and experimental AAA tissues, suggesting it may play a compensatory protective role during the disease process (King et al., 2009; Spin et al., 2011; Doyle et al., 2015). Therefore, we sought to evaluate TGF-β expression in AAA tissues and blood. Western blot analysis revealed a significant increase in TGF-β protein levels in the AAA group compared to the normal group, consistent with gene expression results confirmed by RT-qPCR (Figures 1I–K; Supplementary Figure S2). Additionally, we measured TGF-β concentration in the blood using ELISA. However, our results showed no significant correlation between serum TGF-β levels and the presence or progression of AAA (Figure 1L), consistent with previous findings (Wang et al., 2013). This lack of association may stem from the fact that serum TGF-β levels do not accurately reflect its concentration in aneurysmal tissues or the activity of its downstream signaling pathways.

### O304 suppressed TGF-β-induced VSMC phenotypic switching *in vitro*

**FIGURE 2:**
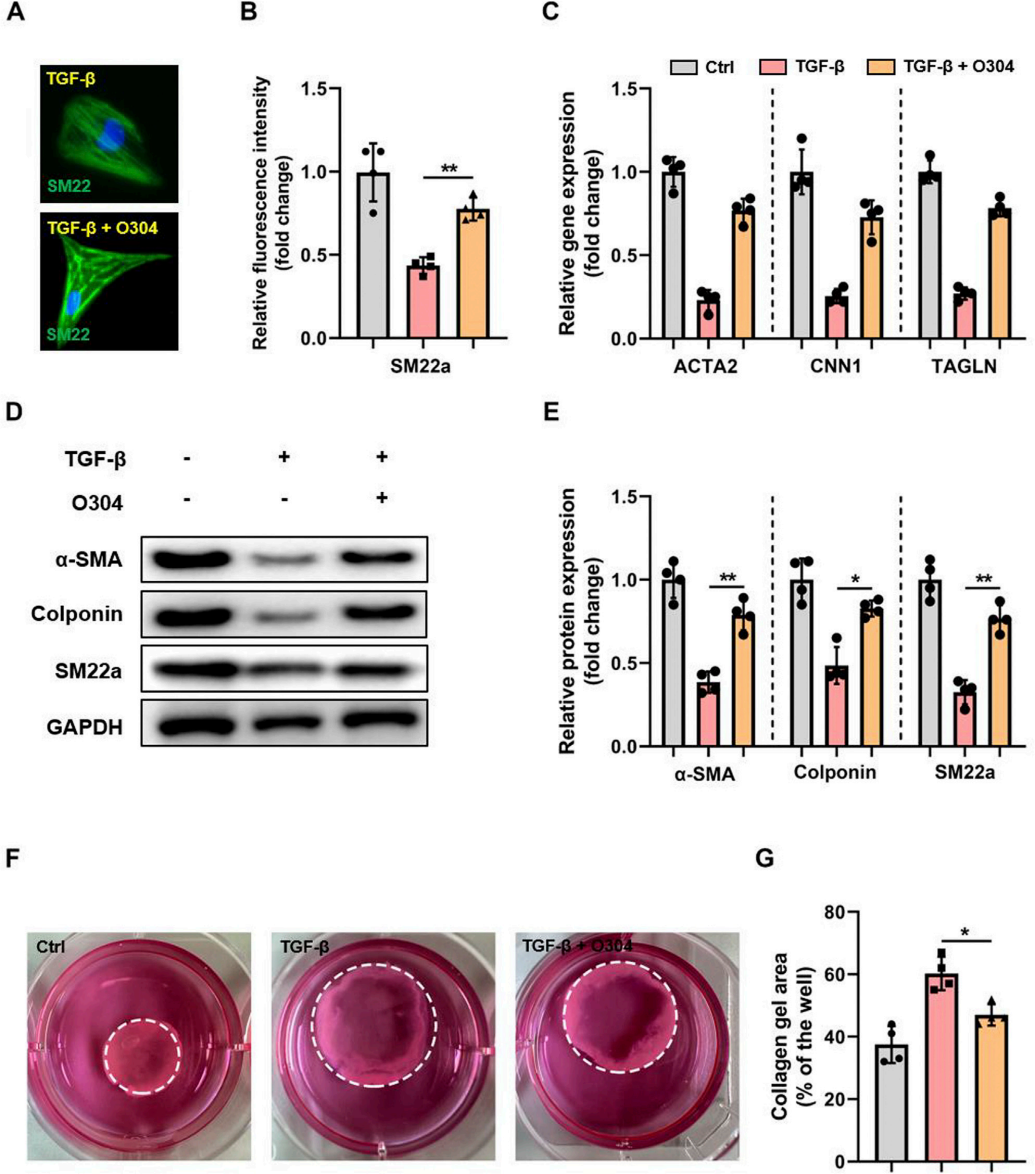
O304 suppressed TGF-β-induced VSMC phenotypic switching through AMPK activation. **(A)** VSMCs were stained with the contractile VSMC marker SM22a, and the nuclei were counterstained with DAPI (bar 100 μm). **(B)** Quantification of SM22a expression. **(C)** RT-qPCR of contractile VSMC genes (*Acta2*, *Cnn1*, *Tagln*) was conducted and normalized to GAPDH. **(D, E)** Western blotting was performed to assess the expression of α-SMA, colponin, and SM22a. **(D)** Representative immunoblots. Full-length blots were presented in Supplementary Figure S1. **(E)** Quantification of protein expression via densitometry analysis and normalization to GAPDH. **(F, G)** VSMCs were digested and resuspended before being mixed with Matrigel in a 1:4 ratio. The resulting mixture was replated into 24-well plates and incubated for 12 h. The contractile ability of VSMCs was assessed by evaluating the area of collagen formation. **(F)** Representative images of VSMCs forming contractile rings. **(G)** Quantitative analysis of the collagen area was performed to measure VSMC contractility. n = 5 independent experiments. Significance was evaluated via one-way analysis of variance followed by Tukey’s *post hoc* test in **(B, D, E, G)** *p < 0.05 and **p < 0.01. AMPK, adenosine monophosphate-activated protein kinase; α-SMA, α-smooth muscle actin; GAPDH, glyceraldehyde-3-phosphate dehydrogenase; RT-qPCR, reverse transcription-quantitative polymerase chain reaction; TGF-β, transforming growth factor-β; VSMC, vascular smooth muscle cell.

To explore the role of the AMPK signaling pathway in VSMC phenotypic switching, we evaluated whether O304, a novel AMPK agonist, could suppress TGF-β-induced VSMC phenotypic switching *in vitro*. Fluorescence microscopy indicated that SM22a (VSMC marker) was significantly decreased in the TGF-β group compared with the control group, and an apparent reversal of this decrease in SM22a was observed in the TGF-β + O304 group (Figures 2A, B). Given that the downregulation of VSMC contractile genes and increased production of inflammatory mediators are markers of VSMC phenotypic switching, we evaluated the expression of inflammation-related genes by RT-qPCR. O304 increased the expression of contractile VSMC genes, including *Acta2*, *Cnn1*, and *Tagln* (Figure 2C). We also measured protein expression via Western blotting (Figure 2D; Supplementary Figure S3). The TGF-β group exhibited downregulated expression of α-SMA, SM22a, and colponin, while the expression of these proteins was increased in the O304-treated group compared with the TGF-β group (Figure 2E). We conducted contractile ring assays to assess the effects of O304 on VSMC contractility to elucidate its regulatory role in VSMC phenotypic switching. VSMC contractility was significantly enhanced in the O304 group compared with the TGF-β group (Figures 2F, G). We also conducted EdU staining to evaluate VSMC proliferation. Consistent with the aforementioned trends, VSMC proliferation was notably enhanced in the O304 group compared with the TGF-β group. Taken together, these results demonstrated that O304 suppressed TGF-β-induced VSMC phenotypic switching *in vitro*, underscoring the role of AMPK signaling in regulating VSMC function.

### O304 increased AMPK/mTOR/MMP protein expression *in vitro*

**FIGURE 3:**
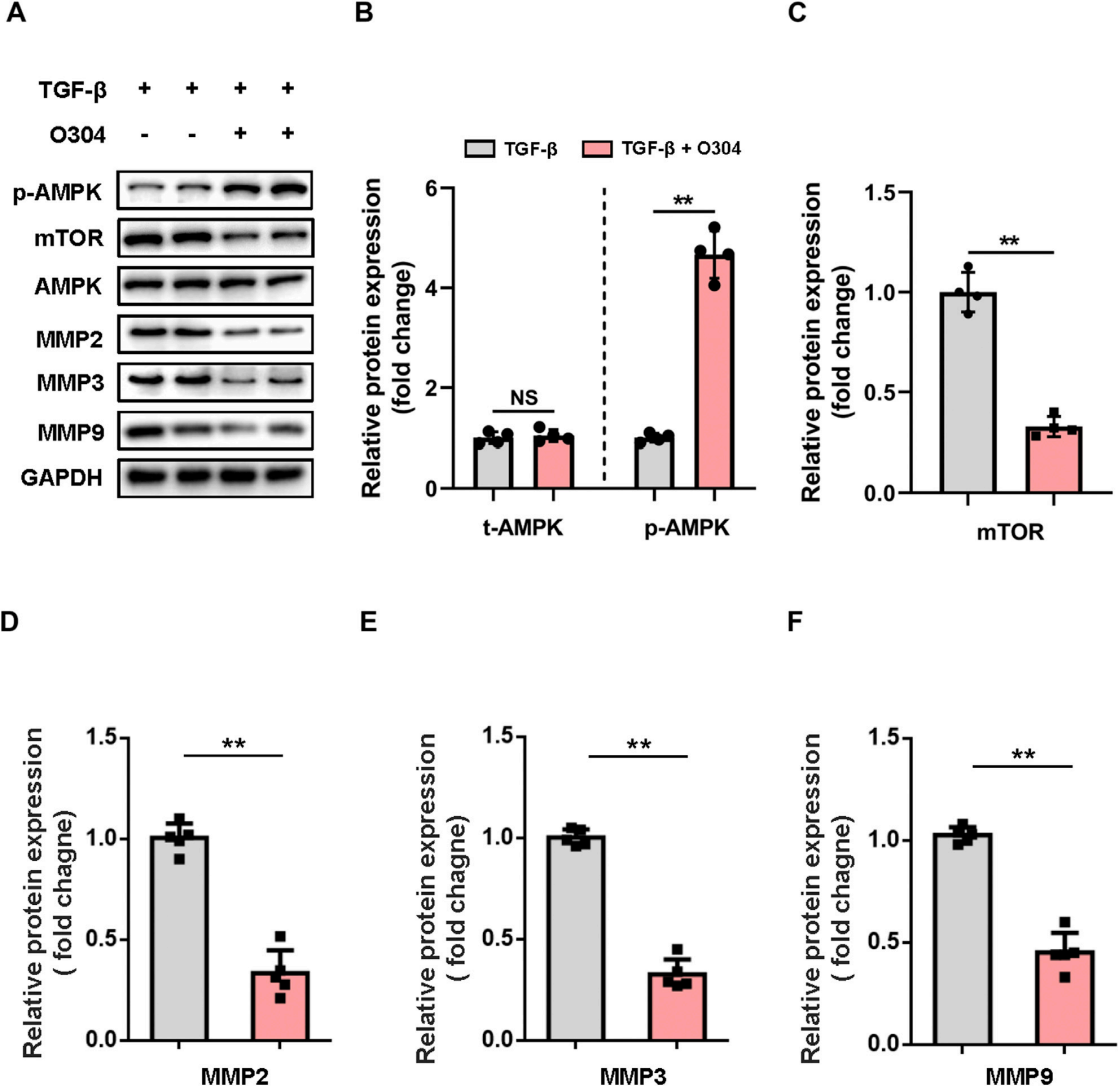
Evaluation of AMPK/mTOR/MMP protein expression *in vitro*. VSMCs were cultured with TGF-β or TGF-β + O304 for 48 h. Protein samples were extracted from the above group for further analysis. **(A, B)** Western blotting was performed to assess the expression of total AMPK and phosphorylated AMPK. **(A)** Representative immunoblots. Full-length blots were presented in Supplementary Figure S2. **(B)** Quantification of protein expression via densitometry analysis and normalization to GAPDH. **(C–F)** Western blotting was performed to assess the expression of **(C)** mTOR, **(D)** MMP2, **(E)** MMP3, and **(F)** MMP9. Quantification of protein expression via densitometry analysis and normalization to GAPDH. n = 4 − 5 independent experiments. Significance was evaluated via one-way ANOVA followed by Tukey’s *post hoc* test in **(B–F)**. *p < 0.05 and **p < 0.01. AMPK, adenosine monophosphate-activated protein kinase; ANOVA, analysis of variance; GAPDH, glyceraldehyde-3-phosphate dehydrogenase; MMP, matrix metalloproteinase; mTOR, mammalian target of rapamycin; VSMC, vascular smooth muscle cell.

To evaluate the impact of O304 on VSMC phenotypic switching, we measured the expression of p-AMPK and mTOR in TGF-β-induced VSMCs. In the O304 group, there was a significant increase in p-AMPK, while the expression of mTOR showed the opposite trend (Figures 3A–C; Supplementary Figure S4). This observation illustrated the positive regulatory effect of O304 on VSMC function and suggested a potential role for the AMPK/mTOR signaling pathway in this process. We next evaluated the MMP family of proteins, including MMP2, MMP3, and MMP9, which are closely associated with cell migration and matrix degradation. Treatment with O304 not only led to AMPK/mTOR signaling pathway activation, but also significantly reduced MMP family protein expression (Figures 3D–F). In addition, we further assessed the expression of MMPs using complementary methods within the TGF-β-induced VSMC phenotypic switching. Just as we expected, our immunofluorescence analysis revealed a pronounced reduction in the expression levels of MMP2 (Supplementary Figures S5A, B), MMP3 (Supplementary Figures S5C, D), and MMP9 (Supplementary Figures S5E, F) following O304 treatment. This suggested that O304 may regulate VSMC function by modulating the expression of MMP proteins. O304 successfully inhibited TGF-β-induced VSMC phenotypic switching by activating AMPK/mTOR/MMP signaling. This discovery not only provides new research avenues for the treatment of arterial diseases, but also lays a solid foundation for further investigation into the molecular mechanisms of O304 and its potential advantages in clinical applications. We anticipate that these findings will provide valuable insights for drug development and therapeutic strategy formulation in future research endeavors.

### O304 treatment inhibited angiotensin II-induced AAA progression

**FIGURE 4:**
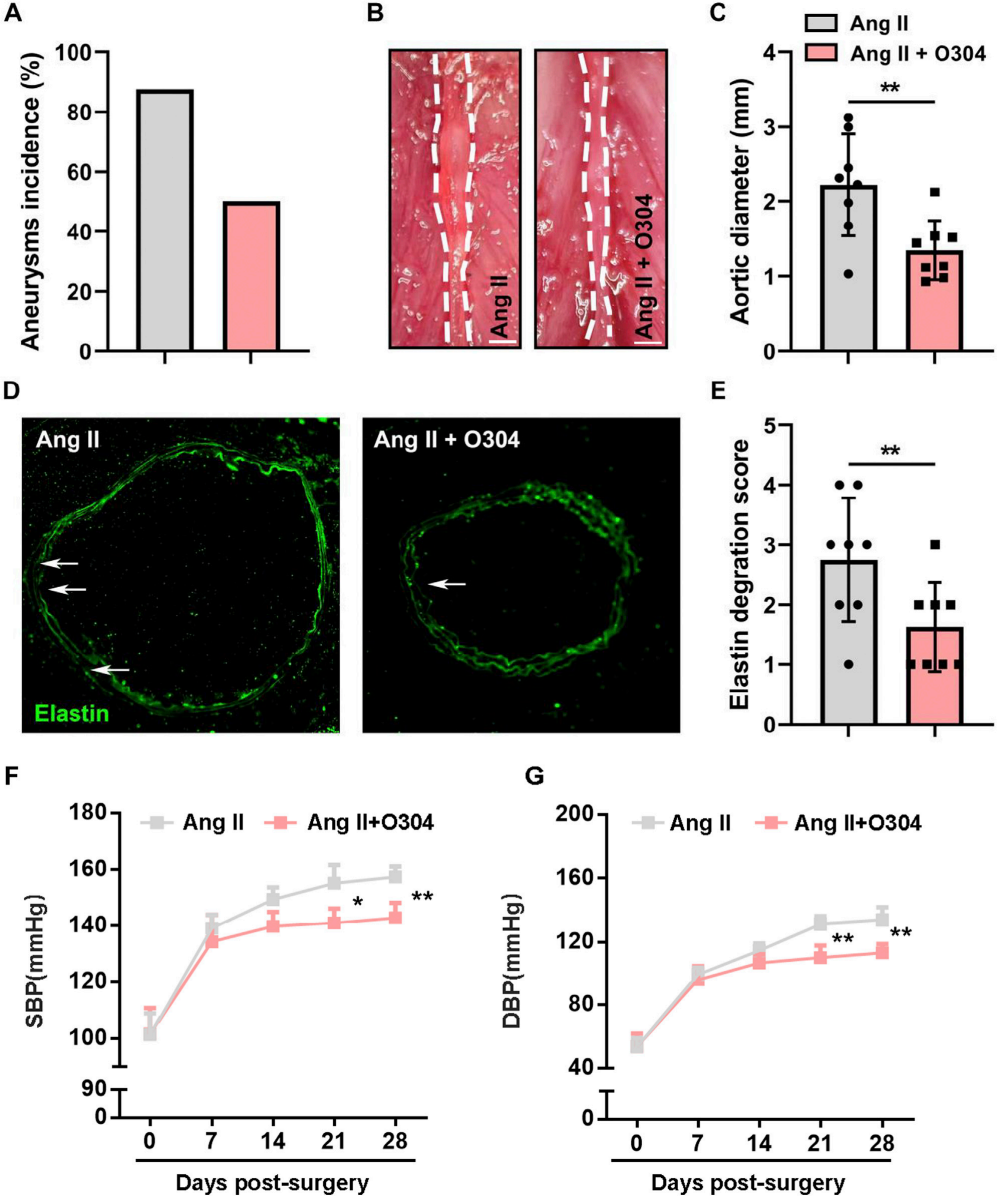
O304 treatment inhibited angiotensin II-induced AAA progression *in vivo*. The mice were divided into the angiotensin II and angiotensin II + O304 groups. **(A)** Quantification of the incidence of aneurysm in each group. **(B)** Representative image of the abdominal aorta on day 28 after surgery. Note: angiotensin II group image in this figure have been re-used from Figure 1A as the control group images. **(C)** Quantification of the aortic diameter. **(D)** Elastin degradation was evaluated through immunofluorescence staining. **(E)** Quantification of the elastin degradation score. **(F, G)** Blood pressure levels were detected on day 7, day 14, day 14, and day 28 after surgery. Quantification of **(F)** Systolic blood pressure (SBP) levels and **(G)** Diastolic blood pressure (DBP) levels. n = 5 independent experiments. Significance was evaluated via the Student’s t-test in **(A, C)** and **(E–G)**. *p < 0.05 and **p < 0.01. AAA, abdominal aortic aneurysm.

To investigate whether treatment with O304 effectively inhibited angiotensin II-induced AAA formation, we constructed a mouse model of AAA and performed weekly measurements of abdominal aortic diameter. The results revealed a significantly lower AAA incidence in mice treated with O304 than in the AAA group (Figure 4A). Specifically, the diameter of the abdominal aorta was markedly reduced in the O304 group following angiotensin II induction compared with the AAA group (Figures 4B, C). This finding demonstrated the effective inhibitory effect of O304 on aneurysm development and provided important clues into the therapeutic mechanism underpinning the effects of O304. Immunofluorescence staining revealed that mice treated with O304 exhibited milder elastic degradation than mice in the control group, suggesting a protective effect of O304 on vascular elasticity and inhibition of AAA development (Figures 4D, E). Moreover, given that hypertension plays a critical role in the development of AAA, we assessed blood pressure both at baseline and following 28 days post-Ang II-induced AAA formation. Our findings showed no significant differences in SBP or DBP between the control and O304-treated groups at baseline (Figures 4F, G). However, as the experiment progressed, O304 treatment led to a marked reduction in both SBP and DBP by day 21, which persisted through day 28 post-surgery (Figures 4F, G). These results suggest a potential antihypertensive effect of O304, which may contribute to its therapeutic role in AAA management. We hypothesized that these effects may be related to AMPK/mTOR/MMP signaling regulation by O304, supporting O304 as a potential therapeutic strategy. Taken together, these results illustrated that O304 inhibited angiotensin II-induced AAA progression in mice.

### O304 suppressed VSMC phenotypic switching but promoted VSMC proliferation in the angiotensin II-induced mouse model of AAA

**FIGURE 5:**
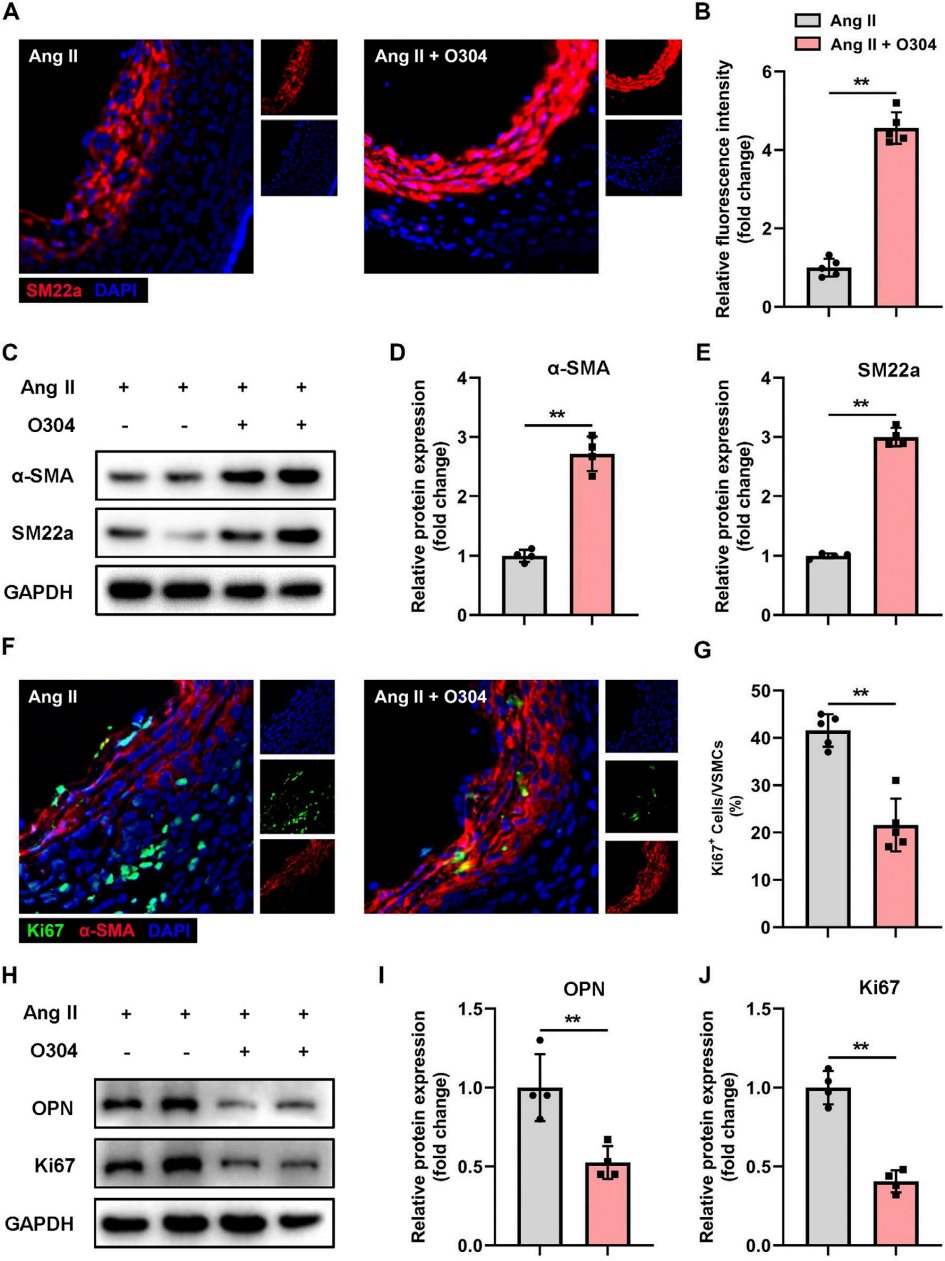
O304 suppressed VSMC phenotypic switching in the mouse model of angiotensin II-induced AAA. The mice were divided into the angiotensin II and angiotensin II + O304 groups. The frozen sections of AAA tissues were collected on day 28 after surgery for further analysis. **(A)** SM22a expression in AAA tissues was detected via immunofluorescence staining. **(B)** Quantification of contractile VSMCs. **(C–E)** Western blotting was performed to assess the protein expression of α-SMA and SM22a. **(C)** Representative immunoblots. Full-length blots were presented in Supplementary Figure S3. Quantification of **(D)** α-SMA and **(E)** SM22a protein via densitometry analysis and normalization to GAPDH. **(F)** AAA tissue samples were stained with anti-α-SMA antibody to identify contractile VSMCs, anti-Ki67 to identify synthetic (proliferative) VSMCs, and DAPI to identify the nuclei. **(G)** Quantification of Ki67^+^ contractile VSMCs. **(H–J)** Western blotting was performed to assess the protein expression of OPN and Ki67. **(H)** Representative immunoblots. Full-length blots were presented in Supplementary Figure S4. Quantification of **(I)** OPN protein expression and **(J)** Ki67 protein expression via densitometry analysis and normalization to GAPDH. n = 4 independent experiments. Significance was evaluated via the Student’s t-test in **(B, D, E, G, I, J)**. *p < 0.05 and **p < 0.01. AAA, abdominal aortic aneurysm; α-SMA, α-smooth muscle actin; GAPDH, glyceraldehyde-3-phosphate dehydrogenase; VSMC, vascular smooth muscle cell; SBP, Systolic blood pressure; DBP, Diastolic blood pressure.

To gain deeper insights into the regulatory role of O304 in VSMC phenotypic switching, we established the mouse model of AAA and thoroughly explored the expression patterns of contractile and synthetic VSMCs in each group. Initially, immunofluorescence staining was performed to detect contractile VSMC markers, revealing a significant increase in SM22a-positive VSMCs in the O304 treatment group on day 28 after AAA induction (Figures 5A, B). This indicated the potential of O304 to inhibit VSMC phenotypic switching from the contractile to the synthetic phenotype. To better understand this effect of O304, Western blotting was conducted. Compared with the AAA group, the expression of contractile VSMC markers, including α-SMA and SM22a, was significantly upregulated in the O304-treated group (Figures 5C–E; Supplementary Figure S10). These findings support the regulatory effects of O304 on the VSMC phenotype. Cell proliferation was analyzed by fluorescence microscopy. In the O304-treated group, the fluorescence intensity of Ki67 suggested a more pronounced decrease in cell proliferation compared with the AAA group (Figures 5F, G). These results suggested that the observed decrease in cell proliferation may have been associated with the ability of O304 to inhibit the transition of VSMCs from the contractile to the synthetic phenotype. Therefore, we evaluated the expression of synthetic VSMC markers and cell proliferation markers by Western blotting. Consistent with the results of immunofluorescence staining, Western blotting showed a significant decrease in the expression of the synthetic VSMC marker OPN and the cell proliferation marker Ki67 in the O304-treated group compared with the AAA group (Figures 5H–J; Supplementary Figure S7). These findings suggested a dual regulatory role of O304 on the VSMC phenotype, inhibiting aberrant transformation, increasing the proportion of contractile VSMCs, and suppressing the proportion of synthetic (proliferative) VSMCs. Based on these findings, we explored the molecular mechanisms by which O304 inhibited VSMC phenotypic switching and proliferation in the angiotensin II-induced mouse model of AAA, providing evidence to support its possible clinical application.

### AMPK/mTOR/MMP signaling contributed to VSMC phenotypic switching

**FIGURE 6:**
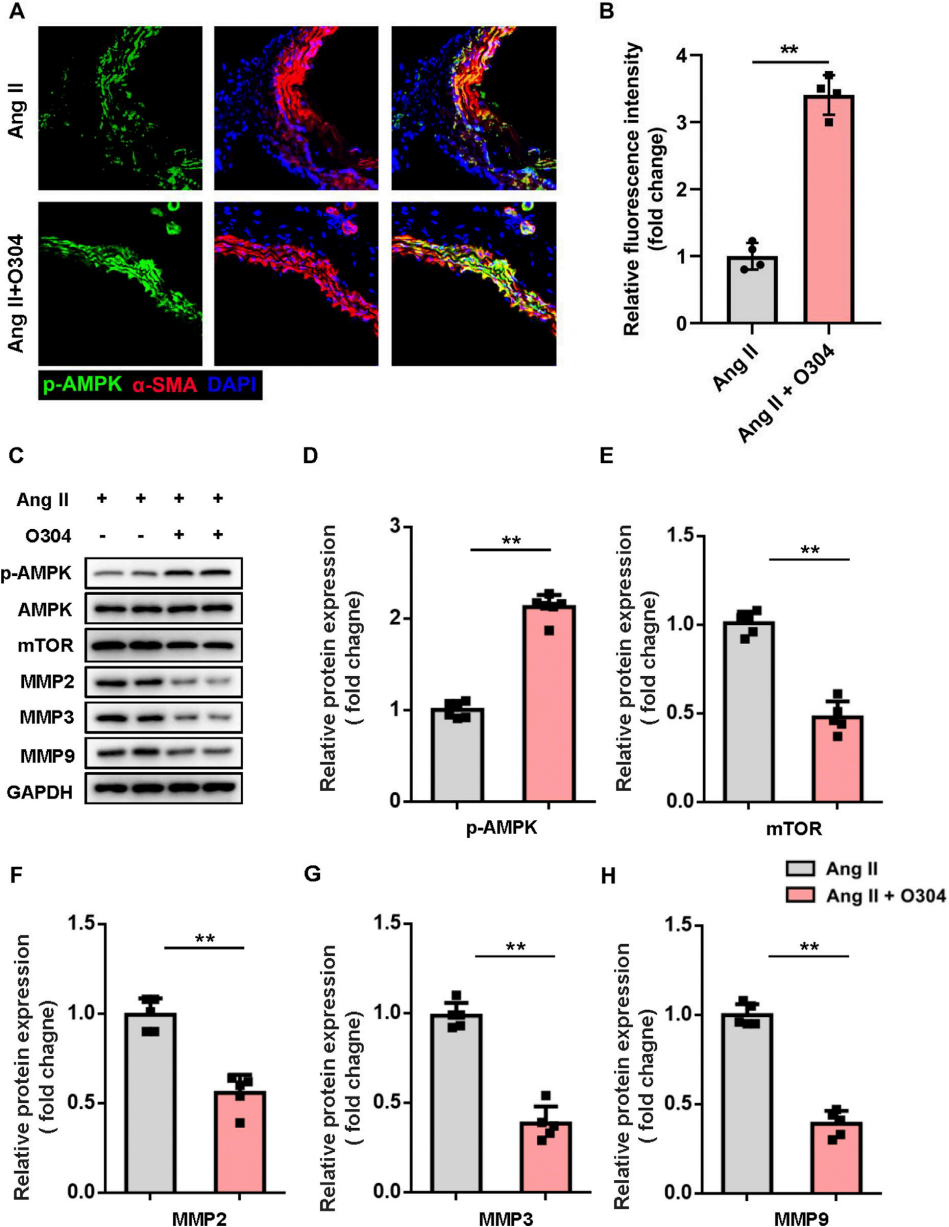
O304 activated AMPK/mTOR/MMP signaling *in vivo*. The mice were divided into the angiotensin II and angiotensin II + O304 groups. Frozen AAA tissue sections were collected on day 28 after surgery for further analysis. **(A)** AAA tissues were stained with antibodies against phosphorylated AMPK (green), α-SMA (red) for contractile VSMCs, and DAPI (blue) for nuclei. **(B)** Quantification of the fluorescence intensity. **(C–G)** Western blotting was performed to assess the protein expression of synthetic VSMCs. **(C)** Representative immunoblots. Full-length blots were presented in Supplementary Figure S3. Quantification of the expression of **(D)** p-AMPK, **(E)** mTOR, **(F)** MMP2, **(G)** MMP3, and **(H)** MMP9, and normalization GAPDH. n = 4-5 independent experiments. Significance was evaluated via Student’s t-test in **(B, D–H)**. *p < 0.05 and **p < 0.01. AAA, abdominal aortic aneurysm; AMPK, AMP-activated protein kinase; ANOVA, analysis of variance; mTOR, mammalian target of rapamycin; MMP, matrix metalloproteinase; RT-qPCR, reverse transcriptase-quantitative polymerase chain reaction; α-SMA, α-smooth muscle actin; VSMC, vascular smooth muscle cell.

In terms of the molecular mechanisms by which O304 inhibited VSMC phenotypic switching and proliferation in the angiotensin II-induced mouse model of AAA, we speculated that the AMPK/mTOR/MMP signaling pathway might play a crucial role. We assessed AMPK signaling pathway activation in contractile VSMCs by immunofluorescence. Compared with the angiotensin II-induced AAA model group, O304 significantly increased AMPK phosphorylation in contractile VSMCs (Figures 6A, B). We subsequently evaluated the expression of key proteins within the AMPK signaling pathway, including p-AMPK, mTOR, and the MMP family (MMP2, MMP3, and MMP9), via Western blot analysis. O304 treatment led to a marked activation of the AMPK pathway, as evidenced by a significant increase in p-AMPK levels compared to the Angiotensin II-induced AAA model group (Figure 6C). This confirms the activation of AMPK signaling by O304. In contrast, the expression of mTOR showed a significant decrease in the O304-treated group (Figure 6D), indicating that the therapeutic effects of O304 may be mediated, at least in part, through the inhibition of mTOR signaling. We also focused on the MMP family, known for its crucial role in VSMC phenotypic regulation. Our Western blot analysis demonstrated a notable reduction in the expression of MMP2, MMP3, and MMP9 following O304 treatment (Figures 6E–G; Supplementary Figure S8). To further validate these findings, we employed complementary methods, specifically immunofluorescence staining, to assess MMP expression in the angiotensin II-induced AAA model. Consistent with our expectations, immunofluorescence analysis revealed a significant suppression of MMP2 (Supplementary Figures S9A, B), MMP3 (Supplementary FigureS S9C, D), and MMP9 (Supplementary Figures S9E, F) in the O304-treated group. These results strongly suggest that O304 modulates VSMC phenotype and may inhibit AAA progression by downregulating the expression of MMP family proteins, which are critical to the pathological remodeling process in AAA. Taken together, these results indicated that O304 participated in VSMC phenotypic switching in the angiotensin II-induced AAA mouse model via AMPK/mTOR/MMP signaling pathway regulation. This finding not only provides a new perspective on the role of O304 in the pathogenesis of AAA, but also offers insights for the future design of therapeutic strategies targeting VSMC phenotypic regulation in AAA.

## Discussion

AAA imposes a significant health and economic burden worldwide. Safe and effective treatment strategies are urgently needed to manage this life-threatening condition (Li et al., 2013; Bor et al., 2015; Etminan and Rinkel, 2017). This study presents novel insights into potential therapies for AAA, producing several key findings. First, O304 inhibited VSMC phenotypic switching from the synthetic to the contractile phenotype. This effect was attributed to AMPK/mTOR/MMP signaling pathway modulation by O304, as elucidated through careful *in vitro* analyses. Second, O304 administration potently inhibited AAA progression and reduced blood pressure. We observed that O304 suppressed angiotensin II-induced AAA progression by activating AMPK/mTOR signaling. This activation was associated with the regulation of MMP2, MMP3, and MMP9 expression, all of which are critical factors in ECM remodeling and modulation of the VSMC phenotype within the aortic wall. These findings underscore the pivotal role of AMPK signaling in AAA pathogenesis. The evidence presented in this study supports the therapeutic potential of targeting AMPK activation using O304, providing a potential avenue for effectively intervening during AAA development.

AAA has a complex pathogenesis that is influenced by multiple molecular and cellular factors. Among them, the dynamic behavior of VSMCs plays a crucial role (Lu et al., 2021; Pan et al., 2022). VSMCs are typically known for their contractile function, but under the influence of microenvironmental stimuli, mechanical stress, and various inflammatory mediators, VSMCs exhibit significant plasticity and can transition between the contractile and synthetic phenotypes. VSMC phenotypic switching from the contractile to the synthetic phenotype is increasingly recognized as a key event in the pathogenesis of AAA (Filiberto et al., 2022). Under normal vascular homeostasis, contractile VSMCs primarily function to maintain vascular integrity, regulate blood flow, and modulate the ECM composition (Ding et al., 2016). However, in pathological conditions, such as chronic inflammation and hemodynamic changes, VSMCs undergo phenotypic switching to the synthetic phenotype. This phenotypic transition is associated with alterations in gene expression and cellular functions.^32^ In the present study, we demonstrated that the transition of contractile VSMCs is one of the most critical pathological processes involved in AAA. Synthetic VSMCs exhibited enhanced proliferation, migration, and ECM remodeling capabilities, which are thought to contribute to vascular remodeling, arteriosclerosis, and aneurysm expansion. Synthetic VSMCs secrete various matrix-degrading enzymes, including MMPs, promoting ECM degradation and weakening the vascular wall, thereby facilitating aneurysm expansion and eventual rupture. Thus, understanding the intricate interplay between inflammatory signals, hemodynamic stress, and VSMC plasticity is crucial for elucidating the pathogenesis of AAA. The mechanisms regulating the VSMC phenotypic transition represent a promising avenue for therapeutic research aimed at slowing AAA progression.

AMPK plays a complex role in cellular energy regulation, metabolic homeostasis, and various biological processes, making it a central player in various disease pathways, including AAA (Kim et al., 2011; Chen et al., 2020). AMPK plays a multifaceted role in AAA, and emerging evidence highlights its potential impact on the pathophysiology of AAA. Downregulation of AMPK activity may lead to changes in cellular function, including inflammation, vascular remodeling, and ECM degradation, thereby affecting AAA development and progression (Yang et al., 2020; He et al., 2021). Furthermore, the role of AMPK in cellular metabolism and autophagy deserves attention in the context of AAA pathogenesis. Dysfunction in cellular energy metabolism and impaired autophagy have been implicated in vascular remodeling and aneurysm formation. AMPK activation has been shown to promote autophagy and maintain cellular energy homeostasis, potentially exerting a protective effect against aneurysm degeneration. However, the exact mechanisms by which AMPK dysregulation contributes to AAA pathogenesis remain to be explored. Although the decrease in p-AMPK in AAA tissues from mice suggests that it may play a role in disease progression, further research is needed to reveal the precise molecular pathways and downstream effectors of AMPK signaling in the context of AAA. Considering the multifaceted functions of AMPK, therapeutic strategies targeting AMPK activation have emerged as potential approaches to mitigate AAA progression (Zhao et al., 2020). Drugs that are capable of restoring AMPK activity or specifically targeting downstream effectors may effectively attenuate inflammation, modulate the VSMC phenotype, and preserve vascular integrity in AAA (Yu et al., 2021). This study may provide valuable insights into the role of O304 in inhibiting VSMC phenotypic switching from the contractile to the synthetic phenotype. Modulation of the AMPK/mTOR/MMP signaling pathway may be a core mechanism through which this is achieved (Chen et al., 2022), whereby O304, through AMPK activation, triggers a series of signaling cascades, ultimately leading to mTOR activation. Activation of mTOR regulates the expression of MMPs, especially MMP2, MMP3, and MMP9, which are crucial for ECM degradation (Ke et al., 2018; Chen et al., 2021). This cascade allows O304 to modulate VSMC function at the molecular level, inhibiting excessive VSMC proliferation and migration and slowing aneurysm progression. This finding provides robust support for further exploration into the potential of O304 as a therapy for AAA (Gandolfi et al., 2011; Gallet et al., 2017).

O304 is a novel pan-AMPK activator that enhances AMPK activity by inhibiting the dephosphorylation of the AMPK α-subunit at Thr172 have demonstrated that O304 offers protective effects in chronic diseases and aging-related conditions (Ericsson et al., 2021; Zhu et al., 2022). For instance, it has been shown to reduce fasting blood glucose levels, improve insulin sensitivity, and enhance peripheral microvascular perfusion, all while lowering blood pressure in animal models and patients with type 2 diabetes (T2D) (Steneberg et al., 2018). Additionally, O304 was found to improve cardiac function and physical capacity in aged mice, indicating its potential to significantly enhance quality of life in elderly populations (Ericsson et al., 2021). Unlike metformin, which modulates AMPK activity indirectly by inhibiting mitochondrial respiration, and thus helps maintain energy homeostasis. Specifically, at millimolar concentrations, metformin inhibits Complex I of the electron transport chain, reducing NADH-driven proton gradients. This suppression lowers ATP production, leading to a decreased [ATP]:[ADP] and [ATP]:[AMP] ratio. As ATP levels fall and AMP and ADP rise, AMPK becomes activated as part of the cellular response to energy stress. This mechanism, while effective, is indirect and dependent on altering the cell’s metabolic state (Miller et al., 2013; Steinberg and Carling, 2019). However, O304 is identified as a pan-AMPK activator that directly increases AMPK activity by preventing the dephosphorylation of AMPK at Thr172. O304 achieves this by specifically inhibiting protein phosphatase 2C (PP2C) from dephosphorylating Thr172, thereby sustaining AMPK activation (Steneberg et al., 2018). Unlike metformin, this mechanism does not rely on reducing cellular ATP levels or altering the cell’s energy balance. Instead, O304 acts directly on AMPK regulation without affecting mitochondrial respiration, which offers a more targeted and sustained activation of AMPK. This distinction highlights the different pathways through which metformin and O304 activate AMPK, with O304 offering a direct and potentially more consistent approach to AMPK activation compared to metformin’s energy-sensing mechanism. This distinct mechanism offers a fresh perspective on O304’s application in AAA therapy, suggesting it may serve as a superior alternative to existing treatment options. However, to date, no specifically investigated the role of O304 in AAA progression. Given the critical role of AMPK signaling in AAA pathology, particularly in the regulation of VSMC phenotypic switching, it is essential to explore the efficacy and safety of O304 as a potential treatment for AAA. In AAA, the phenotypic transition of contractile VSMCs to a synthetic phenotype—marked by downregulation of AMPK signaling components—contributes to disease progression. In our study, we demonstrated that O304 treatment significantly activated the AMPK signaling pathway in both *in vitro* TGF-β-induced VSMC phenotypic switching models and *in vivo* angiotensin II-induced AAA models. This activation was associated with an increase in contractile VSMC markers and a decrease in synthetic markers, suggesting that O304 plays a critical role in modulating VSMC phenotype by reactivating AMPK signaling.

Furthermore, we evaluated the impact of O304 on AAA development *in vivo* and observed that O304 treatment not only inhibited AAA progression but also reduced both systolic and diastolic blood pressure. These results strongly indicate that O304’s therapeutic potential in AAA is mediated through its ability to activate AMPK signaling and regulate VSMC phenotype, which are key processes in AAA pathophysiology. In addition to its effects on VSMC phenotype modulation, O304 treatment significantly reduced the expression of MMPs, including MMP2, MMP3, and MMP9, both *in vitro* and *in vivo*. MMPs are known to play crucial roles in the degradation of the ECM and have been strongly implicated in the progression of AAA. The overexpression of MMPs, particularly MMP2 and MMP9, contributes to ECM breakdown, vessel wall weakening, and subsequent aneurysm formation (Wagenhäuser et al., 2024; Qin et al., 2024). Our findings suggest that the downregulation of MMP expression by O304 may provide an additional mechanism by which this compound exerts its protective effects against AAA development. The reduction in MMP expression following O304 treatment likely contributes to the stabilization of the arterial wall, preventing the excessive ECM degradation that is characteristic of aneurysmal growth. This inhibitory effect on MMPs complements O304’s role in promoting a more contractile VSMC phenotype, creating a dual action mechanism where both cellular and structural components of the vasculature are protected. By simultaneously regulating AMPK signaling, modulating VSMC phenotype, and reducing MMP activity, O304 addresses key pathological processes involved in AAA formation and progression. These findings underscore the multifaceted therapeutic potential of O304 in AAA treatment. The ability to target both VSMC behavior and ECM stability provides a more comprehensive approach to limiting aneurysm growth and rupture, which could be especially beneficial in the treatment of AAA, a disease known for its complexity and multiple contributing factors (Li Y. et al., 2020). While these results are promising, future studies are needed to further clarify the long-term benefits of O304 on MMP regulation and to assess how sustained reductions in MMP activity contribute to AAA stabilization.

Regarding safety, while O304 has been shown to lower blood pressure and improve metabolic and vascular health in previous studies, its safety profile in the context of AAA treatment requires further investigation. Given that hypertension is a major risk factor for AAA, the observed blood pressure-lowering effect of O304 in our study suggests a potential dual benefit for both AAA inhibition and cardiovascular protection. However, long-term studies are necessary to thoroughly assess the safety of O304, particularly concerning its effects on blood pressure regulation and other cardiovascular parameters in the context of AAA treatment.

## Conclusion

In conclusion, our study is the first to demonstrate that O304 treatment can inhibit AAA progression by activating the AMPK signaling pathway and modulating VSMC phenotype. These findings highlight the potential of O304 as a therapeutic agent for AAA, while also underscoring the need for further research to fully elucidate its safety and long-term efficacy in this context. These findings act as a foundation for future translational research aimed at developing more targeted and efficacious treatments for AAA.

## Data Availability

The original contributions presented in the study are included in the article/Supplementary Material, further inquiries can be directed to the corresponding author.
