# Supplementary figures and images for: O304 alleviates abdominal aortic aneurysm formation via AMPK/mTOR/MMP pathway activation
Source: Front Pharmacol. 2024 Nov 29;15:1457817. doi: 10.3389/fphar.2024.1457817 (PMC11637863; doi:10.3389/fphar.2024.1457817)

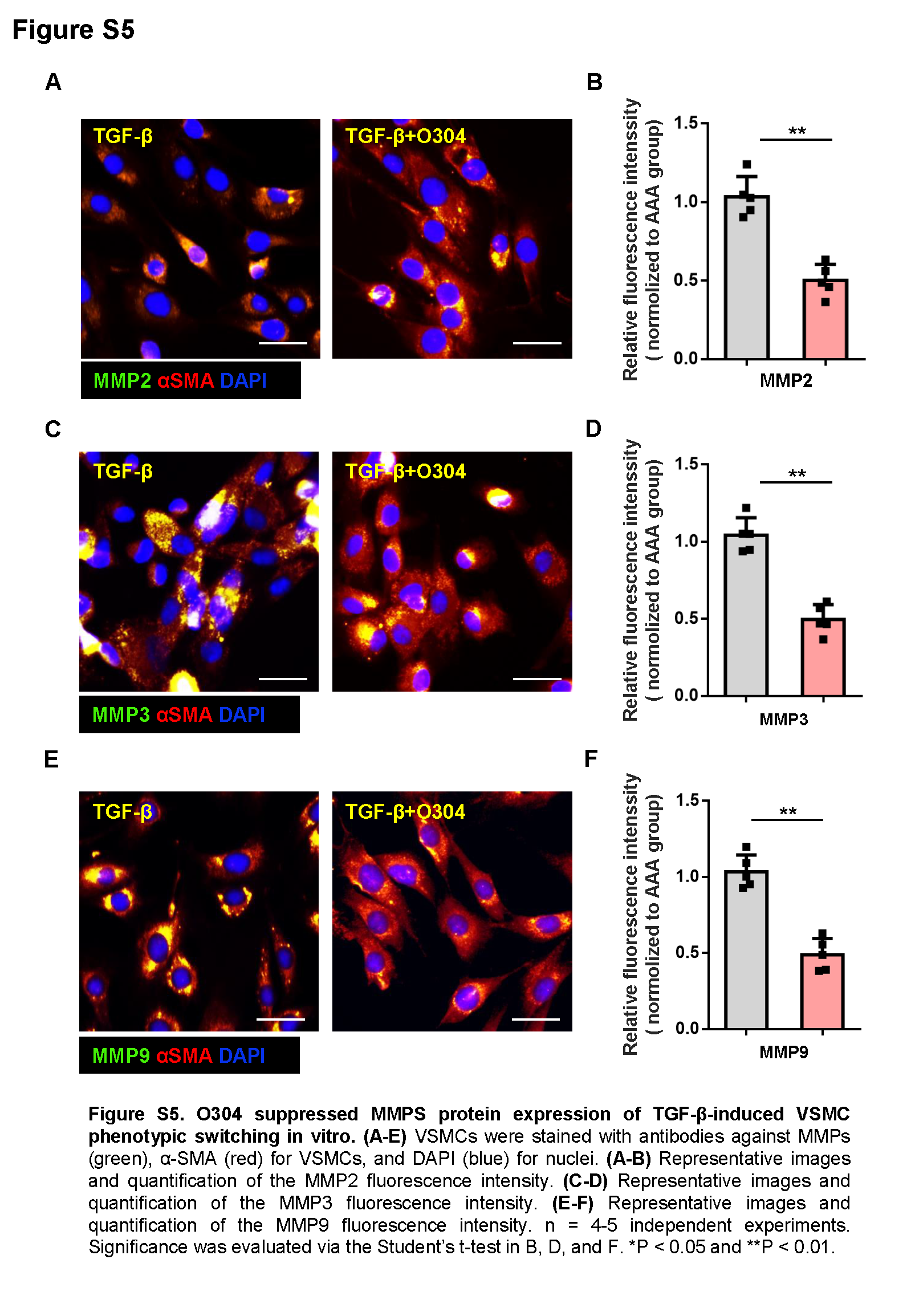

Supplement: Supplementary file 1 [file Image6.tif]

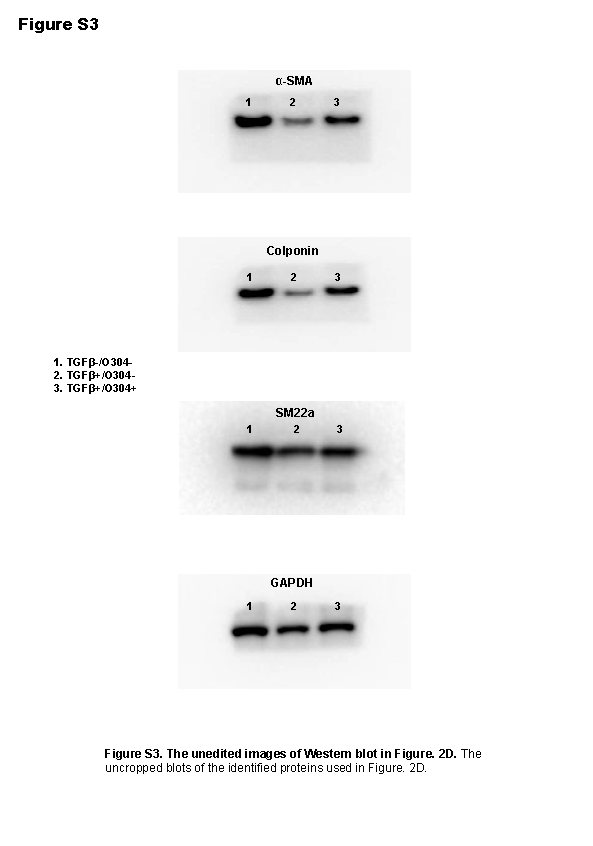

Supplement: Supplementary file 2 [file Image3.tif]

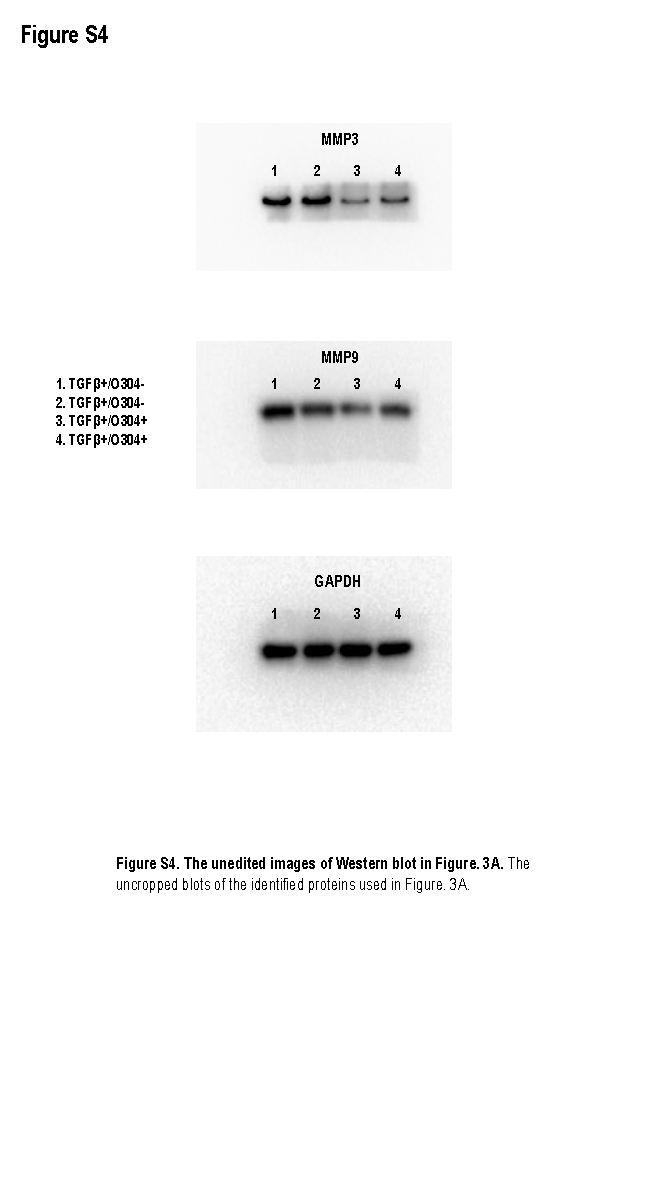

Supplement: Supplementary file 3 [file Image4.tif]

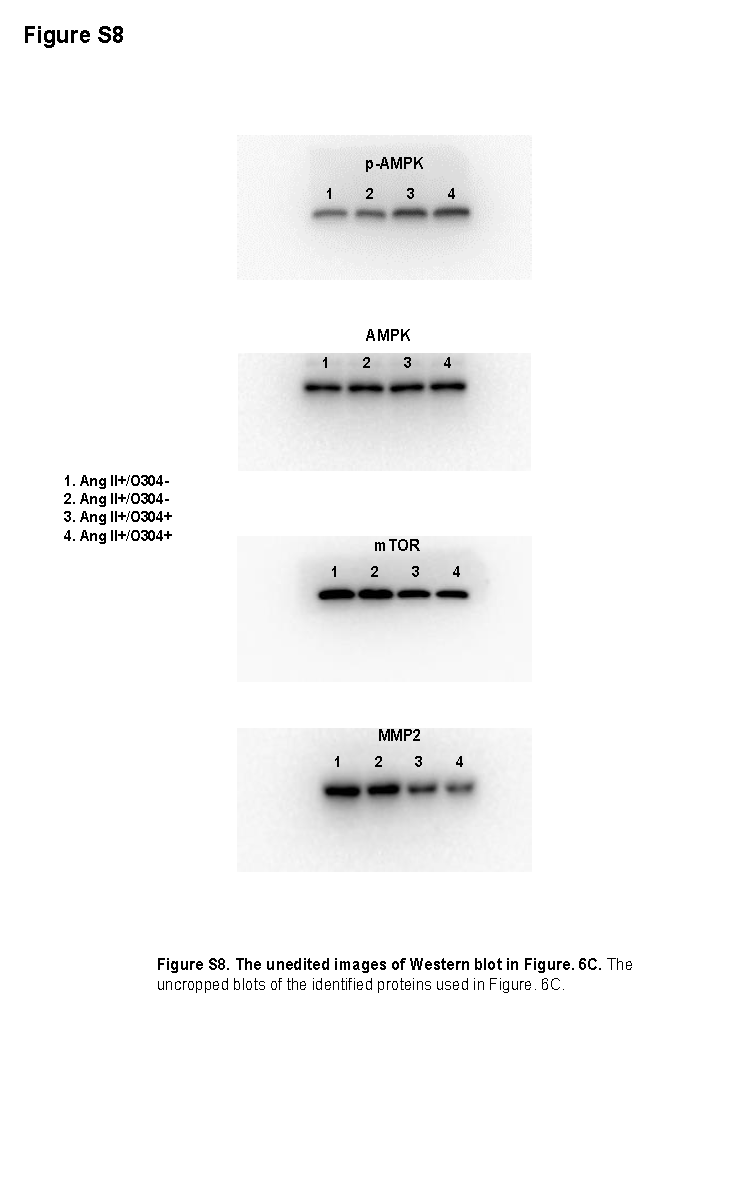

Supplement: Supplementary file 4 [file Image9.tif]

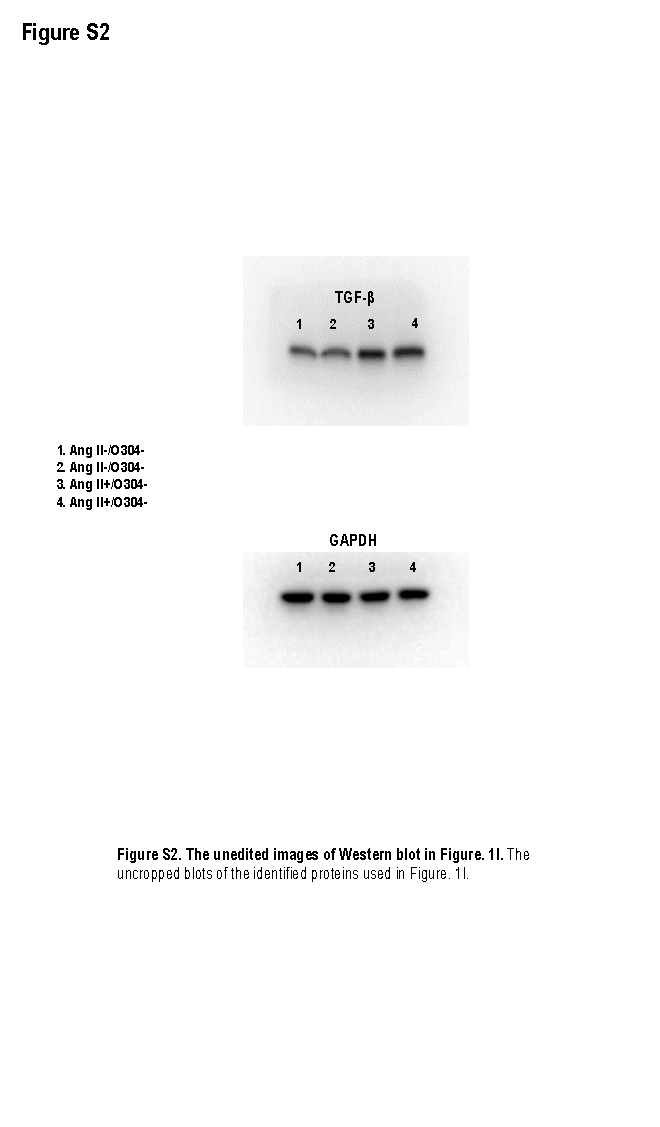

Supplement: Supplementary file 5 [file Image2.tif]

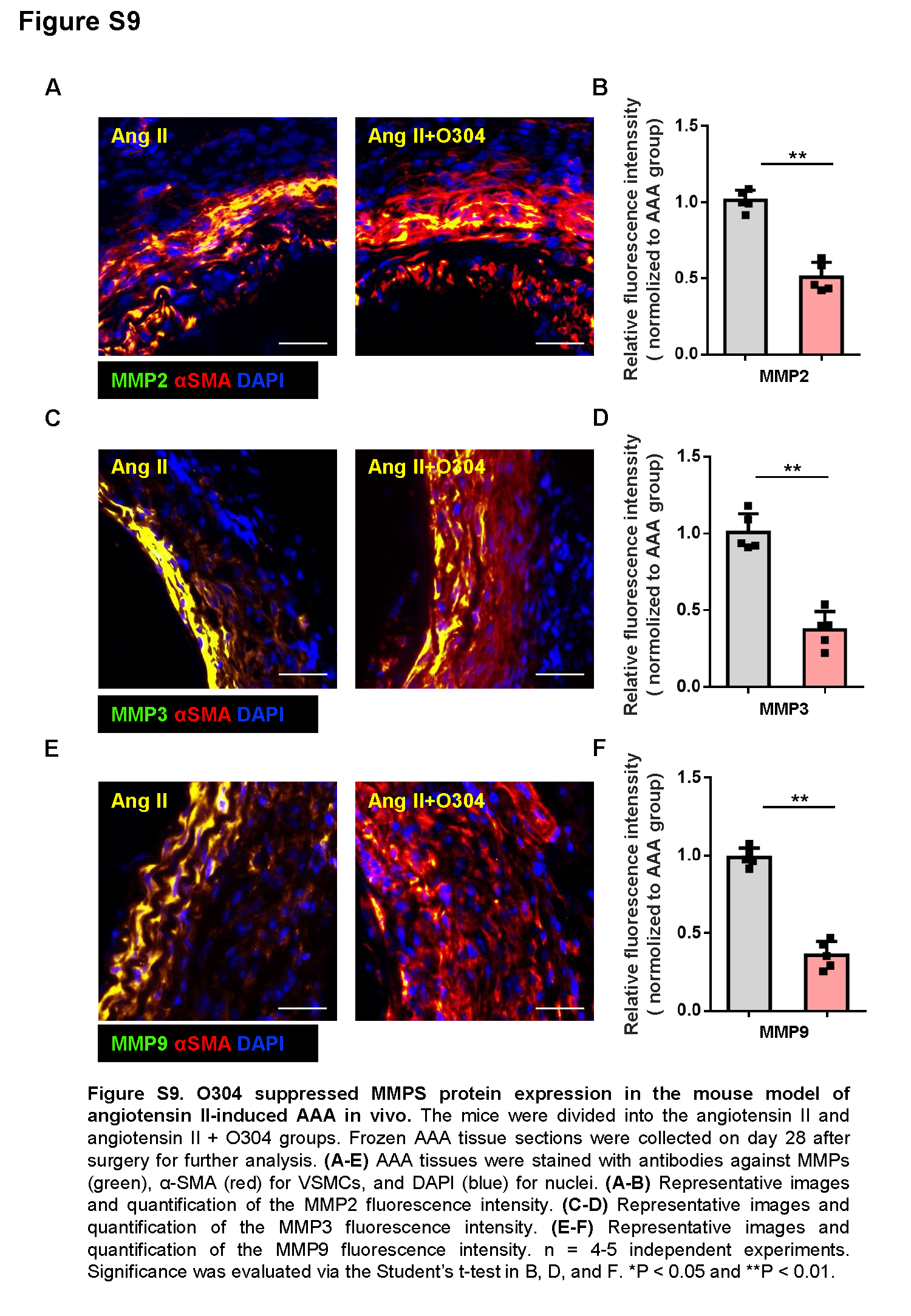

Supplement: Supplementary file 6 [file Image11.tif]

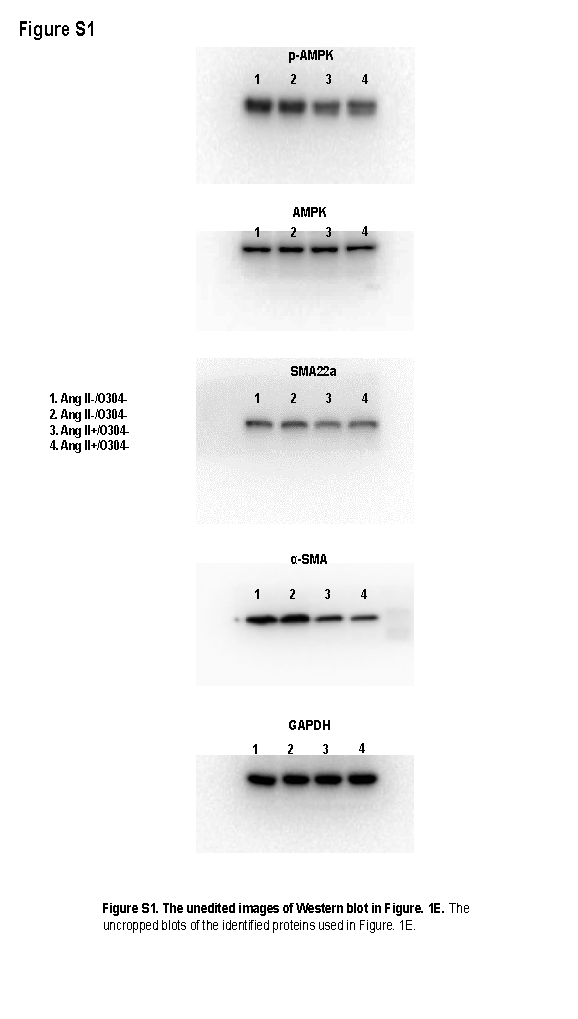

Supplement: Supplementary file 7 [file Image1.tif]

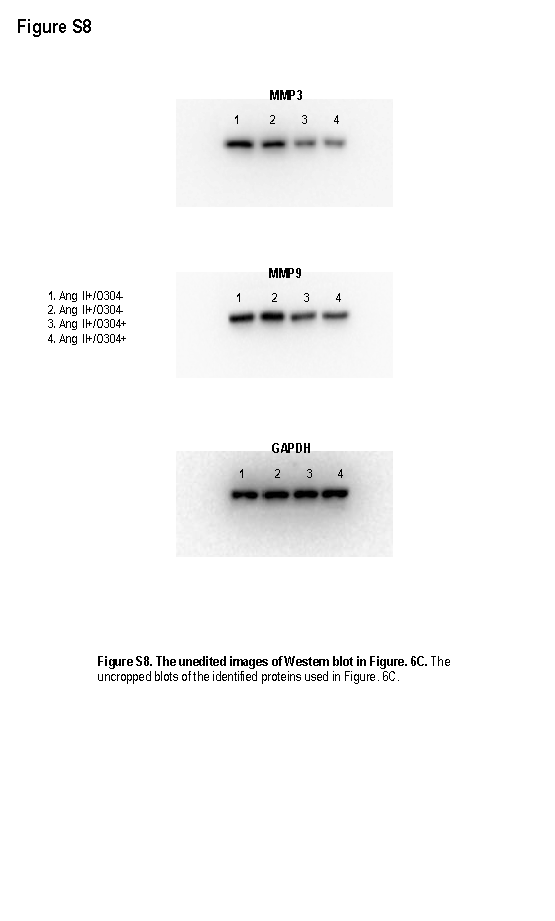

Supplement: Supplementary file 8 [file Image10.tif]

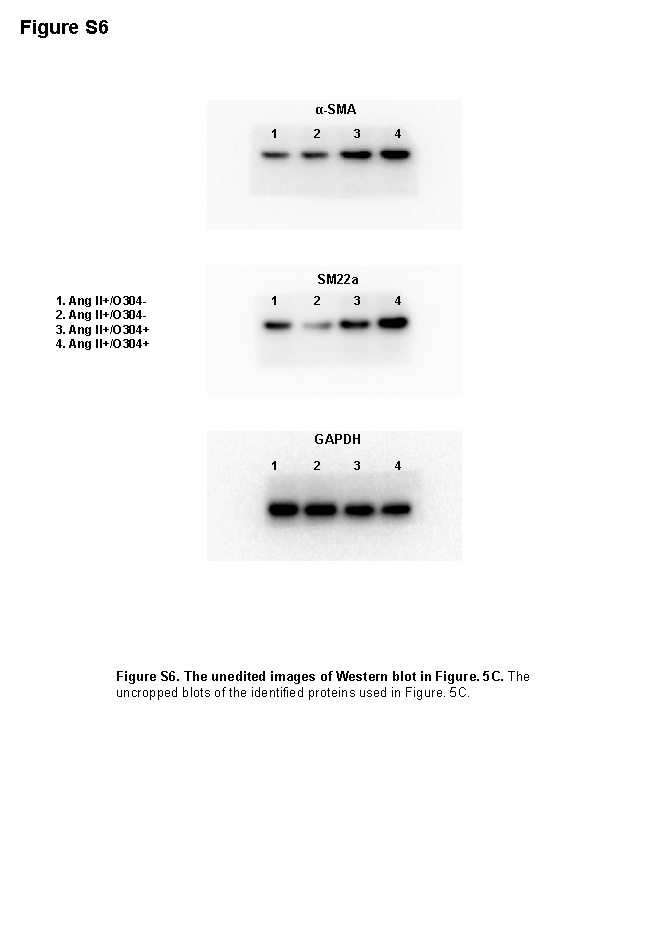

Supplement: Supplementary file 9 [file Image7.tif]

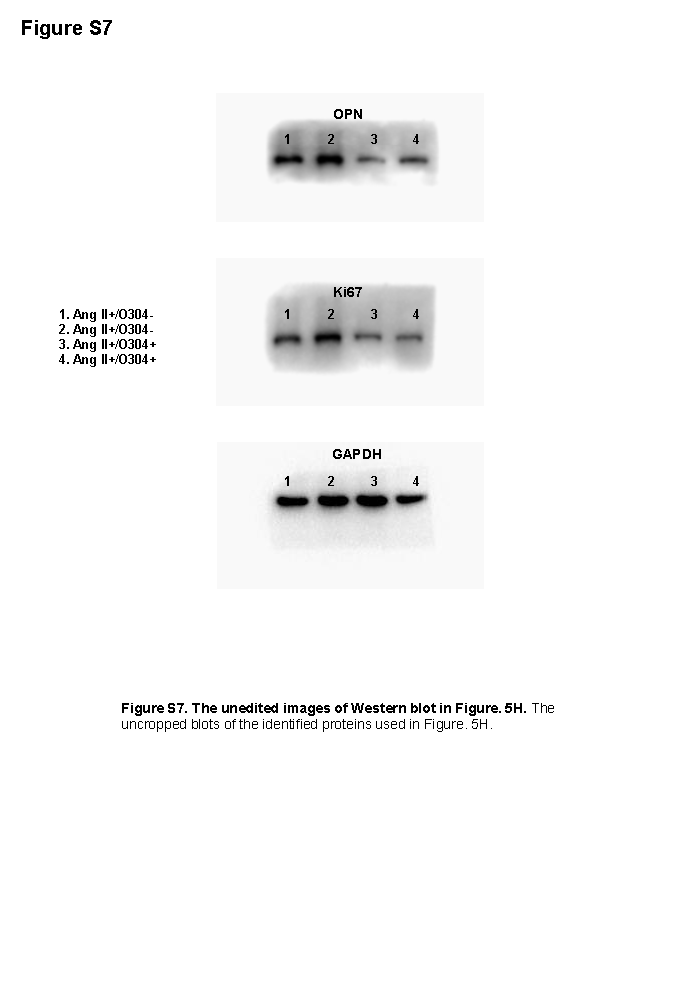

Supplement: Supplementary file 10 [file Image8.tif]

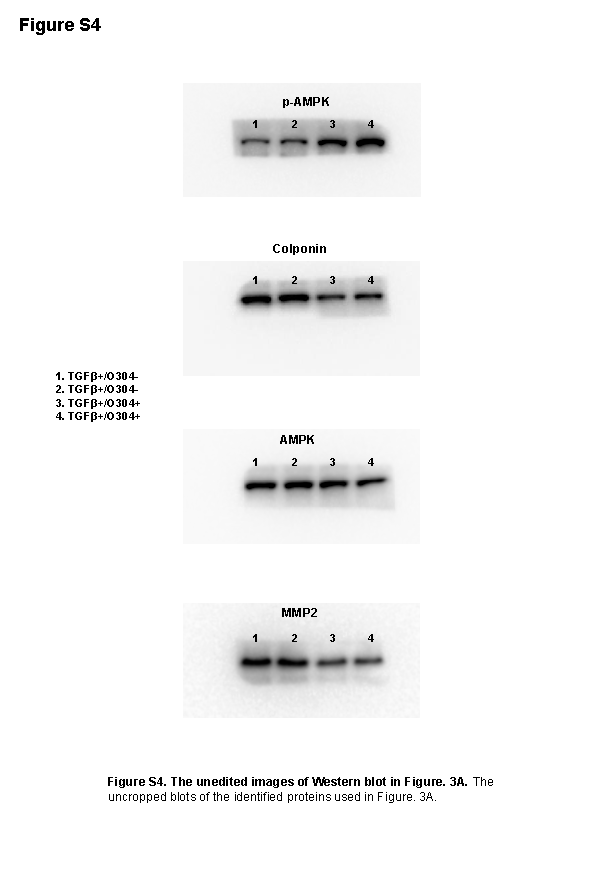

Supplement: Supplementary file 11 [file Image5.tif]
